# Data mining and the evolution of biological complexity

**DOI:** 10.1186/1756-0381-4-7

**Published:** 2011-04-10

**Authors:** Davnah Urbach, Jason H Moore

**Affiliations:** 1 Dartmouth College, Institute for Quantitative Biomedical Sciences, One Medical Center Dr., Lebanon, NH 03756, USA; 2 Dartmouth Medical School, Department of Genetics, One Medical Center Dr., Lebanon, NH 03756, USA; 3 Dartmouth Medical School, Department of Community and Family Medicine, One Medical Center Dr., Lebanon, NH 03756, USA

## 

A common challenge of identifying meaningful patterns in high-dimensional biological data is the complexity of the relationship between genotype and phenotype. Complexity arises as a result of many environmental, genetic, genomic, metabolic and proteomic factors interacting in a nonlinear manner through time and space to influence variability in biological traits and processes. The assumptions we make about this complexity greatly influences the analytical methods we choose for data mining and, in turn, our results and inferences. For example, linear discriminant analysis assumes a linear additive relationship among the variables or attributes while support vector machine or neural network can model nonlinear relationships. Regardless, it is a useful exercise to think about where biological complexity comes from as a way to facilitate the selection of data mining methods. One important theory is that evolution has shaped the complexity of biological systems. More specifically, we introduce here canalization as an evolutionary force in biological systems.

Canalization is broadly defined as the evolution of phenotypic robustness to genetic or environmental perturbations [see for e.g. [1-3]]. Canalization buffers developmental pathways against the tendency for both new allelic variants and environmentally-induced noise to generate suboptimal phenotypes, and thereby ensures the reliability of vital mechanisms such as cognition, glucose metabolism or immune response [4].

Canalization implies a reduction in trait variability [1,3], i.e. in the propensity to vary in response to mutations or environmental changes [5], whereas it leaves genetic variability unaffected, allowing for cryptic genetic variation to accumulate [6]. By repressing the expression of existing genetic variation and of novel mutations, canalization reduces the responsiveness of traits to natural selection [5], and hence their potential to evolve [1,3]. However, if selection for canalization weakens, the building-up of hidden genetic variation likely increases the potential for evolutionary divergence [1,4].

Several molecular mechanisms contribute to canalization [see for e.g. [3]], including redundancy [7] and regulatory genetic interactions [5,8,9]. In the present context, redundancy refers to the compensation for the loss of a gene's activity by one or several alternative genes derived from the same ancestor through gene duplication [7]. The notion of canalization through genetic interactions refers to both the modification of existing interactions and the incorporation of new ones as additional genes enter existing genetic networks [9]. Hence in the former case, robustness is achieved because diverse genetic modules - ranging from single haploid genes to complex genetic networks - can produce virtually identical phenotypes, whereas in the latter, it is achieved by ensuring the robust structure of the genetic networks underlying phenotypes.

Canalization provides a theoretical basis for understanding how evolution shapes the genotype-phenotype relationship. Complexity due to nonlinearity can arise as a result of highly redundant gene networks that evolved to stabilize phenotypes, thus making organisms more resistant to genetic and environmental perturbations. The implications of canalization for data mining could be significant and should be kept in mind when planning, executing and interpreting the analysis of high-dimensional biological data.

## References

[B1] Gibson G Wagner GP Canalization in evolutionary genetics: a stabilizing theory? BioEssays 2000 22 372 380 10.1002/(SICI)1521-1878(200004)22:4<372::AID-BIES7>3.0.CO;2-J 10723034

[B2] Siegal ML Bergman A Waddington's canalization revisted: developmental stability and evolution Proc Natl Acad Sci 2002 99 10528 10532 10.1073/pnas.102303999 12082173 PMC124963

[B3] Flatt T The evolutionary genetics of canalization Quart Rev Biol 2005 80 287 316 10.1086/432265 16250465

[B4] Gibson G Decanalization and the origin of complex disease Nat Rev Gen 2009 10 134 140 10.1038/nrg2502 19119265

[B5] Wagner GP Booth G Bagheri-Chaichian H A population genetic theory of canalization Evolution 1997 51 329 347 10.2307/2411105 28565347

[B6] Waddington CH Canalization of development and the inheritance of acquired characters Nature 1942 3811 563 565 10.1038/150563a0 13666847

[B7] Wilkins AS Canalization: a molecular genetic perspective BiosEssays 1997 19 257 262 10.1002/bies.950190312 9080776

[B8] Rice SH The evolution of canalization and the breaking of von Bear's laws: modeling the evolution of development with epistasis Evolution 1998 52 647 656 10.2307/2411260 28565257

[B9] Proulx SR Phillips PC The opportunity for canalization and the evolution of genetic networks Am Nat 2005 165 147 162 10.1086/426873 15729647

